# The Method of Putting up the Vulcanite Base for Dentures

**Published:** 1859-11

**Authors:** 


### THE METHOD OF PUTTING UP THE VULCANITE BASE FOR DENTURES.

Procure the most perfect impression, then make a light model of good plaster, then adapt to it a common gutta percha plate—this is used to obtain the articulation—then take up the antagonizing models in the usual way, then arrange the teeth upon the gutta percha plate. Bend the pins of the teeth, so that they will hold firmly in the rubber, then build on wax, where it is designed the rubber shall be, dress it smooth. Now place the model containing the teeth and plate in the bottom part of the iron flask, and fill up with plaster round the middle; trim it smooth, then varnish and oil it. Then put on the upper part of the flask, then fill completely with plaster, put on the top, and then with the clamp, screw it all together. After the plaster has set, separate the flask, warm it, and remove the gutta percha plate and wax from the cast and from about the teeth. Then warm the counter model, which retains the teeth, to a hundred and fifty, to one hundred and seventy-five degrees. Then proceed to pack in the rubber, in small pieces, slightly warmed, till the counter mould is more than filled. Now cut channels in either half of the mould, three or four in number, from the mould to the outside of the flask, through which the redundant gum may pass. Now put the flask together, put on the clamp, and screw down gently, make it quite warm, and close the plate perfectly with the clamp. It is now ready to go into the vulcanizing chamber.

